# Reproductive health – a blind spot in psychotherapeutic treatment? Evidence of insufficient consideration of reproductive factors in routine care

**DOI:** 10.1080/19585969.2026.2653598

**Published:** 2026-04-19

**Authors:** Sinha Engel, Jana Saskia Langer, Enno Maaß, Jan Richter

**Affiliations:** a Experimental Psychopathology, University of Hildesheim, Hildesheim, Germany; b Clinical Psychology and Behavioral Health Technology, Friedrich-Alexander-Universität Erlangen-Nürnberg, Erlangen, Germany; c German Psychotherapists Association (DPtV), Berlin, Germany

**Keywords:** Luteal phase, premenstrual phase, follicular phase, ovulation, climacteric, infertility

## Abstract

**Introduction:**

Reproductive health impacts mental health. Consequently, reproductive factors should be assessed in psychotherapy to ensure correct differential diagnosis, precise case conceptualisation and efficient treatment. It has not been analysed to which extent psychotherapists assess reproductive factors in routine care.

**Methods:**

Analyses were based on data from Germany-wide surveys with *n* = 390 psychotherapists and *n* = 291 patients. From their respective perspective, we examined how frequently psychotherapists proactively assessed whether patients experienced a menstrual cycle, menopause, used hormonal contraception, experienced a pregnancy, fertility treatment, or birth, whether these factors were associated with patients’ symptoms, and how relevant they were perceived to be. Further, we examined how psychotherapists acquired knowledge about the reproductive factors and which psychotherapist characteristics increased the likelihood of considering them.

**Results:**

We identified a gap between low proactive assessment and high perceived relevance of reproductive factors. Psychotherapists acquired their knowledge through personal experience rather than clinical training or further education. Greater knowledge and perceived relevance increased the likelihood of proactively assessing reproductive factors.

**Conclusions:**

There is a clear need to incorporate a standardised, proactive assessment of reproductive factors into routine care. The responsibility to disclose them should not be left to the patients. Developing appropriate teaching content and tailored anamnestic instruments can help to overcome the neglect.

## Introduction

Reproductive factors (i.e., factors related to reproduction that are associated with significant hormonal changes and can occur during the female reproductive life span, including experiencing a menstrual cycle, the menopause, using hormonal contraception, as well as experiencing a pregnancy, fertility treatment, or birth (Soares and Zitek 2008; Peters et al., 2023)), should actively be discussed in psychotherapeutic treatment for two main reasons. Firstly, because reproductive factors are highly prevalent. Secondly, because they have a clear and lasting impact on mental health.

Regarding the high prevalence of reproductive factors, a first striking thought is that the life of every individual begins with birth. Admittedly, the active form of giving birth is a more drastic event in adult life– one that 85% of all women^1^ will have experienced by the age of 44 (Schweizer and Graham 2025). Pregnancy and birth significantly increase the risk to develop a mental disorder. The risk of depression is 21% pre- and 17% postnatally (Wang et al. 2021; Yin et al. 2021), the risk of an anxiety disorder is 15% pre- and 10% postnatally (Dennis et al. 2017). About 25% of all births fulfil the criteria of a traumatic event (Blecker et al. 2025) and the risk of childbirth-related PTSD is 5% (Heyne et al. 2022). Further, unintended pregnancies, denied and perceived abortions, miscarriages and fertility treatments are reproductive factors that can put enormous strain on those experiencing them (Klonoff-Cohen et al. 2001; Biggs et al. 2017; Biggs et al. 2015; Kuhlmann et al. 2023).

Starting with the menarche, girls and women are significantly more likely to develop depression than boys and men (Deecher et al. 2008). About 29% of women with a menstrual cycle suffer from the premenstrual syndrome (Borenstein et al. 2003) and 3% from premenstrual dysphoric disorder (Reilly et al. 2024). Among cycling patients with a depressive disorder, 60% experience premenstrual symptom exacerbations (Hartlage et al. 2004). Although hormonal contraception controls hormone fluctuations and their negative impact on psychopathological symptoms (Carlini et al. 2022), it can also increase the risk of depression (Skovlund et al. 2016). The vulnerable reproductive stage comes to an end with menopause, which is often accompanied by an increased risk of depression: Between 45% and 68% of perimenopausal women report elevated depressive symptoms, compared with 28% to 31% of premenopausal women (Maki et al. 2019). Further, menopause is associated with stressful somatic symptoms such as hot flashes and night sweats (Santoro et al. 2021).

Given these significant interactions between reproductive and mental health, a comprehensive assessment of reproductive factors patients are currently or were in the past affected by is required during psychotherapeutic anamnesis. From a differential diagnostic perspective, this is important to distinguish reproductive from other mental disorders. Further, reproductive factors need to be considered in case conceptualisation, including functional analytic approaches, and treatment planning to ensure that treatment is optimised towards the individual patients’ needs within a bio-psycho-social framework of mental disorders (Engel 1977).

Reproductive events are strongly stigmatised (Chrisler 2011; Johnston-Robledo and Chrisler 2020). A first survey among US American psychotherapists showed that only 45% of female and 15% of male therapists addressed the menstrual cycle in discussions with their patients (Rhineheart 1989). In a more recent US American survey, psychotherapists were found to discuss the menstrual cycle with 55% of their cisgender female patients (Alaluf 2024). A current survey among Australian mental health professionals showed that 40% of participants ‘sometimes’ discussed the menstrual cycle as a part of their treatment, 18% discussed if ‘often’ and 8% ‘always’ (Stotz and Brand 2025). These data might indicate a positive trend. However, while they provide some evidence of whether and how often the menstrual cycle is addressed in psychotherapy, important research gaps remain. Firstly, previous surveys exclusively rely on the perspective of psychotherapists, ignoring that of patients. Secondly, they exclusively address the menstrual cycle, ignoring other reproductive factors. Thirdly, they considered the menstrual cycle as an overall topic, but further details on whether the menstrual cycle and other reproductive factors influence patients’ mental and physical symptoms remain unexplored. Lastly, an exploration of factors that increase the likelihood of psychotherapists to actively assess reproductive factors is currently lacking.

The present study fills these gaps. While significant progress has been made in understanding the substantial influence of reproductive factors on mental health, our survey examined to which extent this knowledge is transferred into routine care. We considered the perspectives of both psychotherapists and patients, aiming at…

- Analysing how frequently reproductive factors are proactively assessed in psychotherapy
- Analysing how relevant reproductive factors are perceived to be for psychotherapeutic treatment
- Analysing how extensively and in which contexts psychotherapists have acquired knowledge about reproductive factors.
  We did not aim to test directional hypotheses but to provide, for the first time, descriptive reference data.
- Exploring psychotherapist characteristics (sex, age, perceived relevance, knowledge) that increased the likelihood of proactively assessing reproductive factors

Again, the underlying hypothesis was not directional, but exploratory.

## Methods

### Design and inclusion criteria

We conducted two parallelled Germany-wide online surveys (SoSciSurvey, compliant with EU-GDPR). Eligible participants for the first survey were licenced psychological psychotherapists for adults or adolescents, or persons currently in training for their licence in one of the following, in Germany scientifically accredited approaches: Cognitive behavioural therapy, dynamic psychotherapies (including analytic and depth-psychology based psychotherapy) and systemic psychotherapy. Participants had to work in Germany. The second survey targeted adult patients receiving either ongoing psychotherapeutic treatment in one of the listed approaches, or who had completed treatment in the previous two years.

Participants were recruited via (paid) social media posts, websites, public flyers, and mailing lists. We contacted all members of the German Psychotherapists Association (DPtV), the largest advocacy group for psychotherapists in Germany. Participants were informed about the study purpose as well as collection and management of the anonymised data and provided informed consent. The study was approved by the ethics committee of the department for Education and Social Sciences of the University of Hildesheim (#319).

### Measures

#### Participant information

*All participants* were asked about their age, gender and sex and in which setting (outpatient, day-care or inpatient) their treatment(s) took place.

*Therapists* were further asked whether they already had a licence or were in training, and about their respective psychotherapeutic approach(es). They were also asked to indicate all types of disorders they treated and to estimate the number of psychotherapeutic cases they had completed.

*Patients* were further asked which psychotherapeutic approach their psychotherapist worked with, and for which type of disorder they received treatment.

#### Prevalence of reproductive factors among patients

*Overview of reproductive factors*: Patients were asked whether they were affected by the following reproductive factors during the psychotherapy concerned: menstrual cycle, menopause, hormonal contraception, past/current pregnancy, past/current fertility treatment, past birth. Patients responded in a categorical format (e.g., ‘Do you currently have a menstrual cycle?’/’Did you have a menstrual cycle during your last psychotherapeutic treatment?’, response format: ‘yes’/’no’/’I do not know’/’no response’).

*Detailed assessment*: If patients indicated that they were affected by a reproductive factor, they were subsequently asked about more detailed aspects related to them, including their association with psychological and somatic symptoms. To illustrate this approach, we present methods and main results on detailed aspects related to the menstrual cycle in the main text. Detailed aspects related to the other reproductive factors are presented in supplementary material 1. Concerning the menstrual cycle, we asked patients whether, in their subjective perception, the target symptoms treated in psychotherapy fluctuated during the menstrual cycle, as well as for cycle-related fluctuations of other psychological symptoms and other somatic symptoms, with a categorical response format (‘yes’/’no’/’I do not know’/’no response’).

#### Consideration of reproductive factors in psychotherapy

For *patients*, consideration in psychotherapeutic treatment was determined as each question related to the prevalence of reproductive factors/detailed aspects of these factors (see above, e.g., target symptom fluctuation: ‘Do the symptoms targeted in this psychotherapeutic treatment fluctuate during the menstrual cycle?’) was followed by the question ‘Have you been asked about this during psychotherapeutic treatment?’ (response format: ‘yes’/’no’/’I do not know’/’no response’).

As the prevalence of reproductive factors/detailed aspects was not assessed among *psychotherapists,* they first received information about the prevalence rates of the respective reproductive factors/detailed aspects in the general population (see supplementary material 2). Subsequently, psychotherapists were asked to indicate how frequently they asked patients who can potentially be affected by this factor/detailed aspect about it. Using gender-neutral language throughout our survey, we instructed psychotherapists to rate, for instance: ‘Across all mental disorders you treat, please indicate how often you proactively ask patients of potential childbearing age about the following aspects’. This instruction was followed by specific items that parallelled those of the prevalence part of the survey among patients e.g., ‘I ask whether my patients have a menstrual cycle’, ‘I ask about menstrual cycle-related target symptom fluctuations’ (response format: whole numbers between 0, anchored as ‘never’ and 100, anchored as ‘always’).

#### Relevance for psychotherapy

We asked psychotherapists and patients about the perceived relevance of reproductive factors, given that a patient was affected in a way that is related to adverse mental health. To illustrate this, as a menstrual cycle is generally indicative for good reproductive and mental health, we did not ask about the perceived relevance of the fact that a patient had a menstrual cycle. Instead, we asked about the clinically relevant condition, i.e., an absent menstrual cycle (e.g., amenorrhoea). Likewise, we did not ask about the relevance of birth experiences in general, but of distressing birth experiences, in particular.

The following instructions were given to *psychotherapists*: ‘Across all mental disorders you treat: If a patient is affected by the following factors, how important do you think it is for your psychotherapeutic treatment to know about it?’. Again, this instruction was followed by items parallelling the prevalence part of the survey among patients, e.g., ‘Absence of the menstrual cycle (amenorrhoea)’, ‘Menstrual cycle-related target symptom fluctuations’ (response format: whole numbers between 0, anchored as ‘not important at all’ and 100, anchored as ‘highly important’).

As we asked psychotherapists to rate the relevance of the reproductive factor/detailed aspect on the assumption that their patient was affected by it, we decided to ask exclusively those *patients* who had reported being affected in the prevalence part of the survey about their perceived relevance. For instance, patients with amenorrhoea were presented with the instruction ‘How important is it to you that your psychotherapist knows about the following factors’ and the specific item ‘Absence of the menstrual cycle’, and patients with menstrual cycle-related target symptom fluctuations were presented with the specific item ‘Menstrual cycle-related target symptom fluctuations’ (response format: whole numbers between 0, anchored as ‘not important at all’ and 100, anchored as ‘highly important’).

#### Psychotherapists knowledge

Finally, psychotherapists were asked how much knowledge they gained about the reproductive factors during clinical training, further education, and through personal experience, respectively (response format: whole numbers between 0, anchored as ‘no knowledge’ and 100, anchored as ‘a lot of knowledge’).

### Statistical analyses

Prevalence (patient survey), frequency of proactive assessment (both surveys), relevance (both surveys) and knowledge (psychotherapist survey) of reproductive factors were analysed descriptively. Multiple regression analyses were performed to explore the associations between psychotherapist characteristics and the extent to which they reported to proactively assess each reproductive factor. The psychotherapist characteristics explored were age, sex, perceived relevance of the respective reproductive factor, and knowledge about the respective reproductive factor, gained from clinical training, further education and personal experience, respectively. As the assumption of normal distributed regression errors was violated, bootstrapping with 3000 samples were employed to compute confidence intervals and p-values.

## Results

### Flow of participants and sample description

*Psychotherapists*: Of *n* = 603 individuals who started to fill out the survey, *n* = 414 finished it. Of those, *n* = 407 fulfilled the inclusion criteria. In order to ensure data quality, we asked whether participants believed their data should be used. 15 participants indicated that their data should not be used and *n* = 2 did not answer the question. Data from the remaining *n* = 390 psychotherapists were used for the subsequent analyses.

*Patients*: 439 individuals began to fill out the patient survey and *n* = 315 finished it. Of those, *n* = 308 fulfilled the inclusion criteria. Four participants indicated that their data should not be used and *n* = 13 did not answer the quality check. Data from the remaining *n* = 291 patients were used for analyses.

Both samples were predominantly female. Psychotherapists were of middle, and patients of younger age. The predominant treatment approach was cognitive behavioural therapy and a range of mental disorders was treated, broadly reflecting their prevalence rates. Supplementary material 3 includes a detailed description of both samples.

### Prevalence, proactive assessment and relevance of reproductive factors

**Figure 1.**
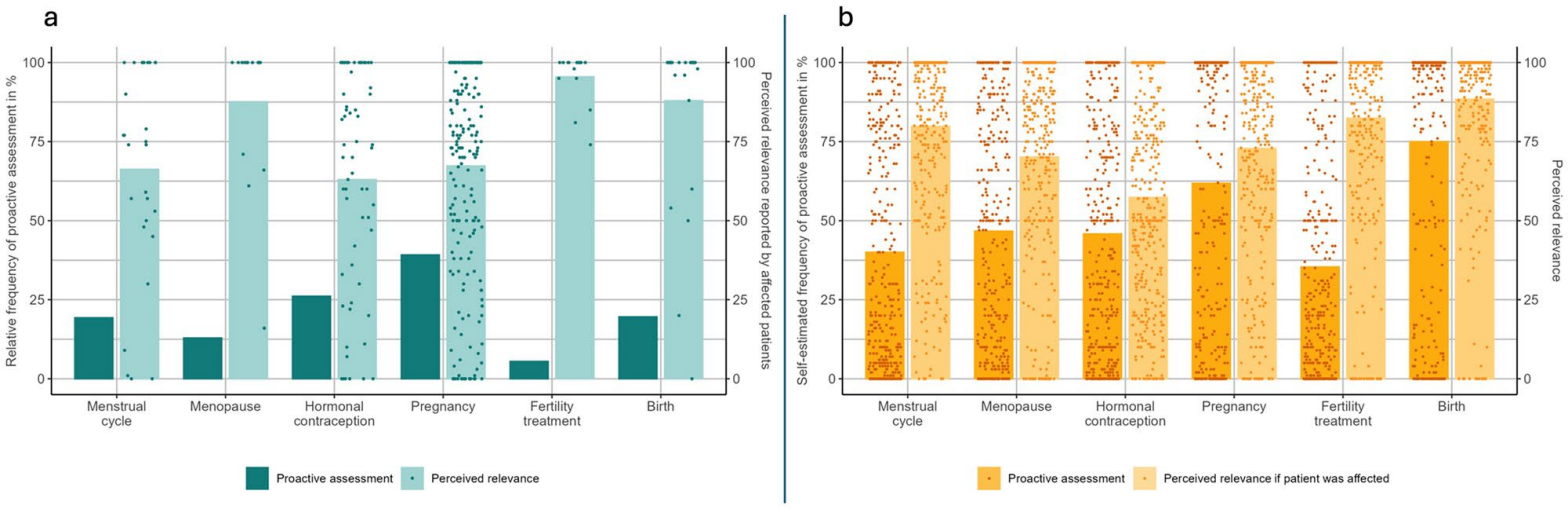
Perspectives from patients (1a, turquoise) and psychotherapists (1b, orange) regarding the frequency of proactive assessment (*n* = 291 patients: yes/no; *n* = 390 psychotherapists: 0 (‘never’) – 100 (‘always’)) and perceived relevance of the reproductive factors in psychotherapy, given that patients are affected (*n* = 28 patients with amenorrhoea, *n* = 15 menopausal patients, *n* = 60 patients using hormonal contraception, *n* = 71 patients with past/current pregnancy, *n* = 16 patients with past/current fertility treatment, *n* = 28 patients with distressing birth experience, *n* = 390 psychotherapists: 0=’not important at all’ – 100=’highly important’).

28 patients were affected by amenorrhoea, *n* = 15 were menopausal, *n* = 60 used hormonal contraception, *n* = 71 had experienced at least one pregnancy, *n* = 16 at least one fertility treatment, and *n* = 28 at least one distressing birth. Figure 1 visualises the descriptively clearly recognisable discrepancies between low proactive assessment and high perceived relevance from the perspectives of patients (panel a) and psychotherapists (panel b).

### Prevalence, proactive assessment and relevance of menstrual cycle-related symptom fluctuations

Concerning menstrual cycle-related symptom fluctuations, 70.38% of patients with a menstrual cycle during psychotherapy (*n* = 260) reported target, 61.15% other psychological and 78.46% other somatic symptom fluctuations. However, only 15.38%, 13.08%, and 11.54% reported that they had been asked about this by their psychotherapist, respectively, as visualised in supplementary material 1. As detailed numerically there, as well, psychotherapists’ estimations of their proactive assessment of menstrual cycle-related symptom fluctuations were also considerably lower than their and the patient’s estimations regarding the perceived relevance.

Regarding the detailed aspects related to the other reproductive factors (see supplementary material 1), the discrepancy between a high number of affected patients and low rates of proactive assessment were especially pronounced regarding menopause-related onset or exacerbation of target, other psychological, or other somatic symptoms. The discrepancy was absent only with regard to live births (presumably, as this information can roughly be equated to the number of children, i.e., standard amnestic information), medical complications and interventions during pregnancy (due to low prevalence rates), and the desire to have one or more (additional) biological children Being affected by a clinically relevant condition of a reproductive factor was consistently rated as highly relevant by patients and psychotherapists.

### Psychotherapists’ knowledge

**Figure 2.**
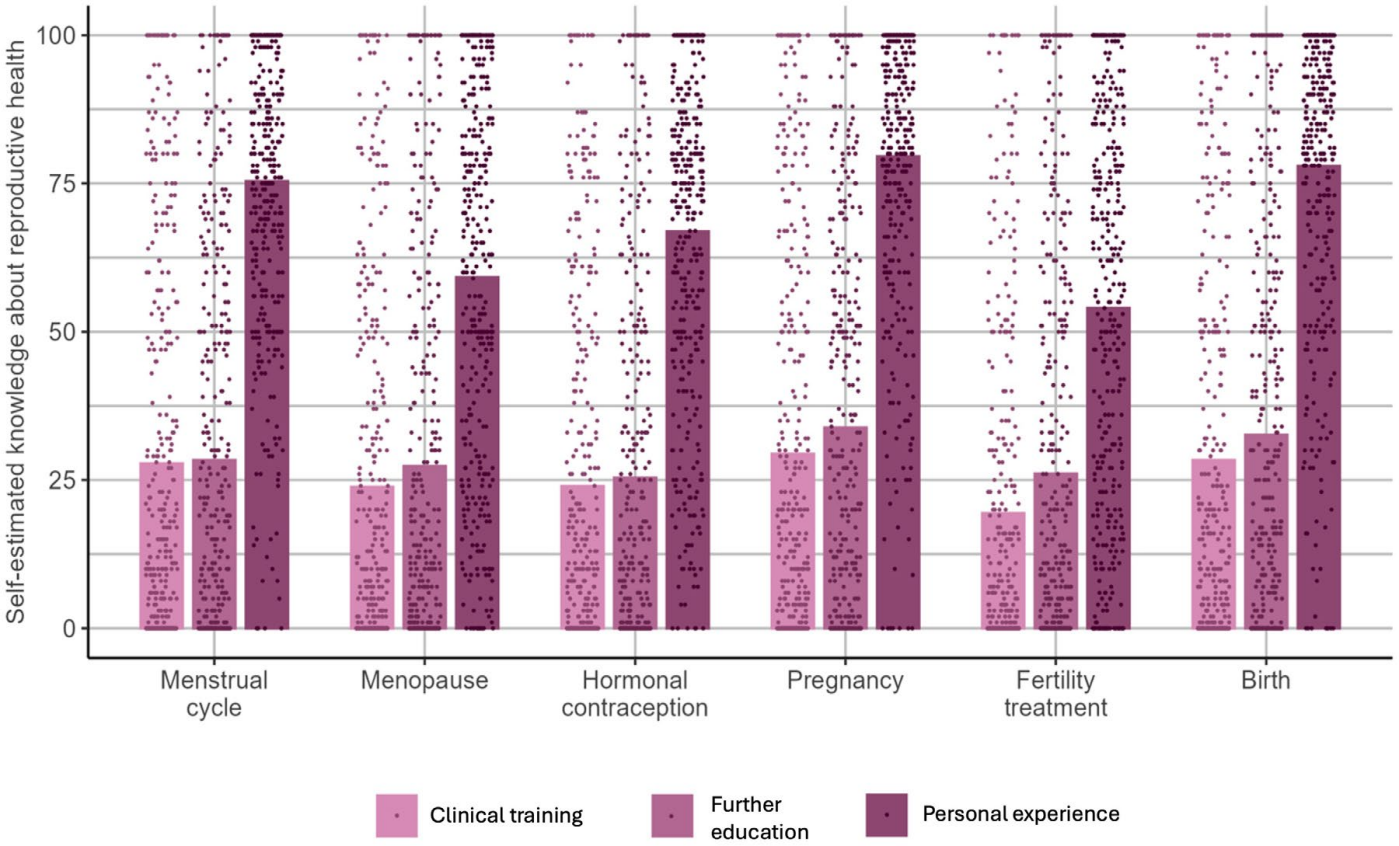
Psychotherapists’ elf-estimated knowledge (0=’no knowledge’ – 100=’a lot of knowledge’) about the respective reproductive factors from clinical training, further education, and personal experience.

As visualised in Figure 2, psychotherapists gained their knowledge about all reproductive factors mainly from personal experience.

### Exploration of factors increasing proactive inquiry

The results of the multiple regression analyses predicting proactive assessment by psychotherapist characteristics are reported in detail in supplementary material 4. As an overall pattern, it shows heterogeneous effects of age and sex. Most consistently, higher perceived relevance was associated with more frequent proactive assessment. Albeit with a more heterogeneous pattern across reproductive factors, more knowledge was also associated with more frequent proactive assessment.

## Discussion

The growing knowledge about the interactions between reproductive and mental health makes it imperative for psychotherapists to actively assess reproductive factors. Due to their potential impact on differential diagnosis, case conceptualisation treatment planning, reproductive factors should be assessed during the initial psychotherapeutic sessions, which are also crucial for establishing a positive working alliance (Wampold and Flückiger 2023). Psychotherapists could even strengthen this alliance by showing that they take a holistic view of their patients, considering the bio-psycho-social determinants of their well-being (Engel 1977). In this study, we comprehensively investigated to which extent the ideal of proactive assessment applied in routine care, taking into account the perspectives of both psychotherapists and patients. Primarily, we identified a strong gap between low proactive assessment, but high perceived relevance of the reproductive factors and their impact. Numerous studies have emphasised the high prevalence of reproductive events and their significant impact on mental health before (Howard et al. 2025), and this is also reflected in our data: The high number of 70.38% of patients reporting that their target symptoms fluctuate during the menstrual cycle aligns well with previous evidence of premenstrual symptom exacerbations (60% of cycling women with a depressive disorder (Hartlage et al. 2004), 41–79% of cycling women with a panic disorder (Kaspi et al. 1994; Cook et al. 1990)). Therefore, the high relevance that psychotherapists and patients attribute to reproductive factors seems appropriate, while the low assessment rates do not. Based on an initial assessment of the reproductive factors, psychotherapists and patients could develop an individual model of how the respective reproductive factors influence the respective symptoms. Again, bio (e.g., hormone sensitivity)-psycho (e.g., subjective meaning)-social (e.g., stigma) processes can be taken into account (Engel 1977), which in turn, can inform possible interventions.

Furthermore, we observed high variance in the answers given by psychotherapists and patients across their ratings of proactive assessment and relevance. From an optimist point of view, this indicates that for some psychotherapists, the proactive assessment of reproductive factors is already part of their routine work. From a more realistic point of view, however, it can be expected that the intersection between the 5.50% of patients who received fertility treatment (to provide just one example) and the 5.50% who were asked about it is very small, if existent at all. This raises concerns that reproductive factors and their impact on patients’ symptoms remain largely unrecognised. Again, optimists might suggest that patients whose symptoms are influenced by reproductive factors would bring this up on their own. However, there are two strong arguments for not leaving this responsibility to patients. Firstly, many patients experience shame related to reproductive factors (Kvalem et al. 2024; Johnston-Robledo et al. 2007). Secondly, patients might not be aware that their symptoms are influenced by reproductive factors. For a formal PMDD diagnosis according to the DSM-5, daily, prospective assessments of PMDD symptoms across two subsequent menstrual cycles are required (American Psychiatric Association 2013). That’s because identifying menstrual cycle-related symptom patterns is challenging and can only be achieved through extensive and precise observation – a task that patients should not be expected to do on their own initiative.

Our study also provides indications as to how the practice of proactive assessment can be promoted. Both, perceived relevance and, albeit with a more heterogeneous pattern, knowledge, were associated with frequency of proactive assessment. Consequently, if psychotherapists are to be trained in assessing reproductive factors, such training should not only provide knowledge about reproductive health, but also emphasise its impact on mental health and provide concrete examples on how to consider reproductive factors therapeutically. The development and evaluation of such treatment programs represents another gap identified by this study, which showed that currently, most knowledge about reproductive factors is gained through psychotherapists’ personal experience. Next to specific training, the development and evaluation of standardised instruments might help psychotherapists to incorporate the assessment if reproductive factors in their daily practice.

The most relevant limitation of this study is a risk of selection bias. We did not recruit representative samples, and it can be expected that our calls for participation appealed to individuals who were already interested in the topic. Concerning the patient sample, this might imply that we primarily recruited patients whose symptoms were indeed affected by reproductive factors, therefore overestimating their impact. However, while our patients indeed frequently reported that reproductive factors impacted their symptoms, these data align well with previous evidence (Hartlage et al. 2004; Kaspi et al. 1994; Cook et al. 1990). Concerning the psychotherapist sample, even if we primarily recruited such interested in and educated about reproductive health, this would mean that in a representative sample, frequencies of proactive assessment and knowledge might even be lower than assumed by our study. However, it is an open question to what extent our results would generalise if we had recruited more diverse samples (e.g., in terms of age, location outside of Germany, and other psychotherapeutic approaches). In the future, this question might be answered by utilising representative recruitment strategies. Next to selection bias, social desirability and recall bias– which differentially affects patients, who were asked to rate the perceived relevance for their actual therapeutic experience, and psychotherapists, who were asked to rate it under the hypothetical scenario that their patient was affected – limit our results. To confirm them, future studies might apply methods that are less prone to these biases, such as chart reviews.

Keeping these limitations and opportunities for future studies in mind, this study demonstrated a strong gap between low proactive assessment, but high perceived relevance of reproductive factors in psychotherapeutic treatment. This indicates a need to better incorporate standardised, proactive assessment of reproductive health into routine care, as the responsibility to disclose reproductive factors and their impact on symptoms should not be left to the patients. Psychotherapists’ knowledge about reproductive health, which currently mainly stems from personal experience, could be promoted through targeted courses during psychotherapeutic training, and standardised assessment could be supported by specific instruments.

## Supplementary Material

Supplemental Material

Supplemental Material

Sup3_2025_11_29.docx

Sup2_2025_07_19.docx

## Note

1 the term ‘woman’ is scientifically inaccurate, as it is unclear whether it corresponds with female sex and/or gender (Peters et al. 2023). Not all individuals who identify as women are affected by the reproductive factors investigated, likewise, neither do all affected identify as women. Therefore, we only use the term ‘woman’ when it was used in original references that we cite.
