# Supplementary material for: Reproductive health – a blind spot in psychotherapeutic treatment? Evidence of insufficient consideration of reproductive factors in routine care
Source: Dialogues Clin Neurosci. 2026 Apr 19;28(1):157–65. doi: 10.1080/19585969.2026.2653598 (PMC13094238; doi:10.1080/19585969.2026.2653598)
Supplement: Supplemental Material [file TDCN_A_2653598_SM7935.docx]

**Supplementary material 4**

**Impact of psychotherapist characteristics on proactive inquiry**

**Table 1**

|  |  | Menstrual Cycle | Menopause | HC | Pregnancy | Fertility Treatment | Birth |
| --- | --- | --- | --- | --- | --- | --- | --- |
| Age | | -0.21 | −0.25 | −0.39* | 0.46 | −0.21 | 0.33 |
|  |  | [-0.58, 0.17] | [−0.65, 0.14] | [−0.61, −0.02] | [−0.27, 0.56] | [−0.57, 0.16] | [-0.04, 0.7] |
| Sex | | -2,27 | −1.61 | −5.98 | 1.42 | −5.13 | 1.2 |
|  |  | [-10.15, 6.31] | [−9.12, 5.68] | [−12.61, 0.9] | [−8.56, 10.43] | [−12.86, 2.76] | [−7.7, 8.95] |
| Perceived relevance | | 0.18* | 0.46** | 0.56** | 0.41** | 0.27** | 0.19 |
|  |  | [0.04, 0.32] | [0.33, 0.6] | [0.46, 0.66] | [0.27, 0.56] | [0.1, 0.43] | [-0.26, 0.42] |
| Knowledge from | | |  |  |  |  |  |
|  | clinical training | 0.05 | 0.13* | 0.07 | 0.13* | 0.22** | 0.1 |
|  |  | [−0.06, 0.16] | [0.02, 0.24] | [-0.04, 0.17] | [0.001, 0.25] | [0.1, 0.34] | [-0.02, 0.21] |
|  | further education | 0.32** | 0.2** | 0.2** | 0.13* | 0.35** | 0.08 |
|  |  | [0.2, 0.44] | [0.09, 0.31] | [0.1, 0.31] | [0.01, 0.27] | [0.24, 0.46] | [−0.04, 0.19] |
|  | personal experience | 0.32** | 0.3** | 0.23** | 0.004 | 0.16** | −0.1 |
|  |  | [0.15, 0.49] | [0.17, 0.43] | [0.11, 0.34] | [−0.18, 0.18] | [0.06, 0.27] | [−0.24, 0.07] |

*Notes.* The table shows the standardized ß, with 95% confidence intervals in square brackets. * indicates *p* <.05. ** indicates *p* < .01. HC= hormonal contraception.
