# Supplementary material for: Reproductive health – a blind spot in psychotherapeutic treatment? Evidence of insufficient consideration of reproductive factors in routine care
Source: Dialogues Clin Neurosci. 2026 Apr 19;28(1):157–65. doi: 10.1080/19585969.2026.2653598 (PMC13094238; doi:10.1080/19585969.2026.2653598)
Supplement: Supplemental Material [file TDCN_A_2653598_SM7934.docx]

**Supplementary material 1**

**Detailed assessment and results on the menstrual cycle, menopause, hormonal contraception, pregnancy, fertility treatment and birth**

**Menstrual cycle – illustration of the results reported in the main text**


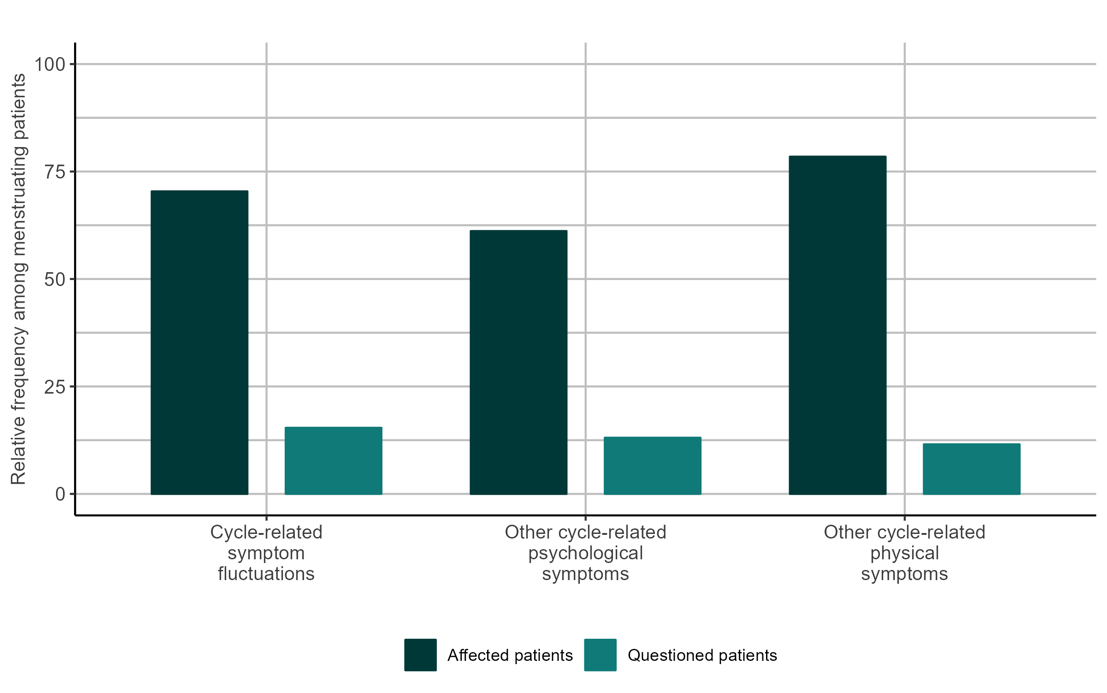


Supplementary figure S1. Discrepancy between number of patients affected by cycle-related fluctuations of target symptoms, other psychological, and other physical symptoms, versus patients asked about this.

**Menstrual cycle**

*Detailed methods: assessment of further menstrual cycle-related aspects*. Next to the menstrual cycle-related symptom fluctuations reported in the main text (with results visualized in supplementary figure S1), we asked whether the target symptoms treated in psychotherapy first occurred or worsened in relation to the menarche, for other psychological symptoms and other somatic symptoms in relation to the menarche, as well as for menstrual cycle regularity and length, The response format was categorical (usually ‘yes’/’no’/ ‘I do not know’/ ‘no response’; except for menstrual cycle length: ‘less than 25 days on average’/ ‘between 25 and 35 days on average’/ ‘more than 35 days on average’/ ‘I do not know’/ ‘no response’).

*Detailed results: prevalence, consideration and relevance of further menstrual cycle-related aspects.* Concerning menstrual cycle-related symptom fluctuations, *n*=183 (70.38%) of patients with a menstrual cycle during psychotherapy (*n*=260) reported target, *n*=159 (61.15%) other psychological and *n*=204 (78.46%) other somatic symptom fluctuations. However, only *n*=40 (15.38%), *n*=34 (13.08%) and *n*=30 (11.54%) reported that they had been asked about this by their psychotherapist, respectively. Psychotherapists proactively asked *M*=51.65% (*SD*=36.86, 0-100) of their patients of potential childbearing age about menstrual cycle-related fluctuations of target symptoms, *M*=43.39% (*SD*=37.12, 0-100) about fluctuations of other psychological symptoms and *M*=38.47% (*SD*=36.47, 0-100) about fluctuations of other somatic symptoms, respectively. Contrasting these numbers, fluctuations of target (affected patients: *M*=85.23, *SD*=20.80, 0-100; psychotherapists: *M*=76.25, *SD*=28.78, 0-100), other psychological (affected patients: *M*=84, *SD*=22.62, 0-100; psychotherapists: *M*=72.11, *SD*=29.53, 0-100), and other somatic (affected patients: *M*=63.77, *SD*=30.8, 0-100; psychotherapists: *M*=60.51, *SD*=31.81, 0-100) symptoms were considered as highly relevant for psychotherapy.

Among patients without primary amenorrhea (*n*=290), *n*=53 (18.28%) reported menarche-related onset or worsening of target symptoms, *n*=71 (24.48%) reported menarche-related other psychological symptoms and *n*=135 (46.55%) reported menarche-related somatic symptoms. However, only *n*=9 (3.1%), *n*=10 (3.45%), and *n*=14 (4.83%), respectively, reported that they had been asked about this by their psychotherapist. Data from psychotherapists indicated that they proactively asked *M*=22.24 (*SD*=31.56, 0-100), *M*=32.7 (*SD*=35.43, 0-100) and *M*=32.22 (*SD*=36.32, 0-100) of their patients of potential childbearing age about menarche-related target, other psychological and somatic symptoms, respectively. However, patients and psychotherapists considered it as important for the psychotherapist to know about an association between menarche, the psychotherapeutic target symptoms (affected patients: *M*=73.02, *SD*=27.71, 0-100; psychotherapists: *M*=65.54, *SD*=33.81, 0-100), other psychological symptoms (affected patients: *M*=65.97, *SD*=31.29, 0-100; psychotherapists: *M*=61.99, *SD*=33.83, 0-100), and other somatic symptoms (affected patients: *M*=49.5, *SD*=31.8, 0-100; psychotherapists: *M*=54.24, *SD*=34.97, 0-100).

Among patients with a menstrual cycle during the psychotherapy concerned (*n*=260), n=55 (21.15%) suffered from menstrual cycle irregularities, even though only *n*=40 (15.38%) were asked about this by their psychotherapists. Psychotherapist-rated frequency of proactively asking about menstrual cycle irregularities was *M*=36.04% (*SD*=37.34, 0-100). At the same time, menstrual cycle irregularities were also perceived as relevant for psychotherapy (affected patients: *M*=55.35, *SD*=30.75, 0-100; psychotherapists: *M*=54.16, *SD*=32.09, 0-100).

Among patients with a menstrual cycle during the psychotherapy concerned, *n*=30 (11.54%) and *n*=28 (10.77%) stated that their cycle was overly long and overly short, respectively. *n*=15 (5.77%) of the patients reported that their psychotherapist has asked about menstrual cycle length, and psychotherapists indicated that they proactively asked *M*=19.46% (*SD*=30.66, 0-100) of their patients of childbearing age about menstrual cycle length. However, overly long and short menstrual cycles were also perceived as relevant for psychotherapy (patients with overly long menstrual cycles: *M*=46.9, *SD*=29.49, 0-100; patients with overly short menstrual cycles: *M*=50.33, *SD*=33.95, 0-100; psychotherapists: *M*=47.84, *SD*=32.55, 0-100).

**Menopause**

*Detailed methods: assessment of menopause-related aspects.* Concerning the menopause, we asked affected patients whether the target symptoms treated in psychotherapy first occurred or worsened during the menopausal transition, as well as for other psychological symptoms and other somatic symptoms related to the menopausal transition (response format: ‘yes’/’no’/ ‘I do not know’/ ‘no response’). Analogous to the methodology described in the manuscript, we asked patients whether they had been proactively been asked about these aspects (response format: ‘yes’/’no’/ ‘I do not know’/ ‘no response’) and we asked psychotherapists how often they proactively asked about these aspects (response format: whole numbers between 0, anchored as ‘never’ and 100, anchored as ‘always’). We asked both participant groups about the perceived relevance of these aspects for psychotherapy (response format: whole numbers between 0, anchored as ‘not important at all’ and 100, anchored as ‘highly important’).

*Detailed results: prevalence, consideration and relevance of menopause-related aspects.* As noted in the manuscript, *n*=15 (48.39%) of patients aged >= 41 years stated that they were affected by the menopause. Of those, *n*=8 (53.33%) reported an association between the menopause and their target symptoms, *n*=4 (26.67%) reported an association with other psychological and *n*=9 (60%) with other somatic symptoms. However, only *n*=1 (6.67%), *n*=1 (6.67%) and *n*=2 (13.33%) were asked about target, other psychological and other somatic symptoms related to the menopause, respectively. Psychotherapists indicated that they asked *M*=46.57% (*SD*=38.68, 0-100), *M*=45.88% (*SD*=38.81, 0-100), and *M*=43.85% (*SD*=38.65, 0-100) of their patients with a uterus who are or can soon be expected to be beyond childbearing age, about an association between menopause and target, other psychological and other somatic symptoms, respectively. At the same time, both groups of participants considered it as important for the psychotherapist to know if a patient was affected by menopause-related onset or worsening of target symptoms (affected patients: *M*=91.25, *SD*=24.75, 30-100; psychotherapists: *M*=73.53, *SD*=32.57, 0-100), other psychological symptoms (affected patients: *M*=91.5, *SD*=17, 66-100; psychotherapists: *M*=69.16, *SD*=33.36, 0-100) and other somatic symptoms (affected patients: *M*=94.56, *SD*=8.2, 78-100; psychotherapists: *M*=59.43, *SD*=34.21, 0-100), respectively.


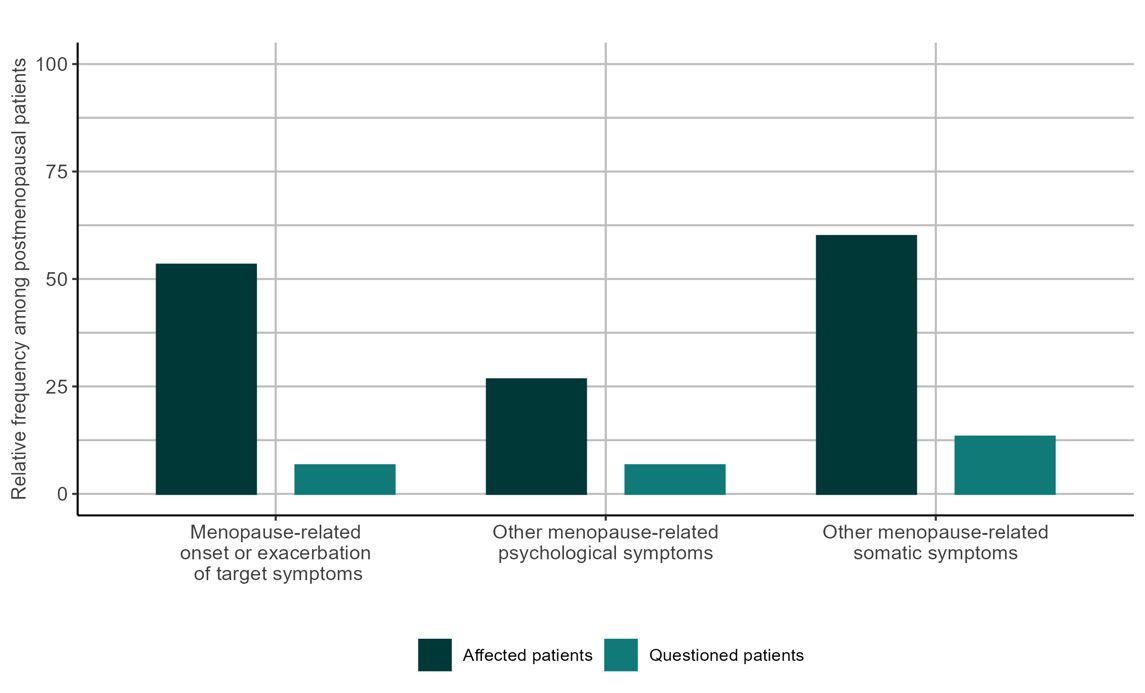


Supplementary figure S2. Discrepancy between number of patients affected by menopause-related onset or worsening of target symptoms, other psychological, and other physical symptoms, versus patients asked about this.

**Hormonal contraception**

*Detailed methods: assessment of hormonal contraception-related aspects*. We asked patients who reported that they used hormonal contraception during psychotherapy whether the psychotherapeutic target symptoms first occurred or worsened while starting hormonal contraception, as well as for other psychological symptoms and other somatic symptoms related to hormonal contraception (response format: ‘yes’/’no’/ ‘I do not know’/ ‘no response’). Analogous to the methodology described in the manuscript, we also asked patients whether they had been proactively been asked about these aspects, we asked psychotherapists how often they proactively asked about these aspects, and we further asked both participant groups about the perceived relevance of these aspects for psychotherapy.

*Detailed results: prevalence, consideration and relevance of hormonal contraception-related aspects.* As noted in the manuscript, *n*=60 (20.62%) patients stated that they used hormonal contraception during psychotherapy. Of those, *n*=10 (16.67%) reported an association between the use of hormonal contraception and their target symptoms, *n*=15 (25%) reported an association with other psychological and *n*=26 (43.33%) with other somatic symptoms. Only *n*=10 (16.667%), *n*=11 (18.33%) and *n*=9 (15%) were asked about target, other psychological and other somatic symptoms related to hormonal contraception, respectively. Psychotherapists asked *M*=37.67% (*SD*=36.64, 0-100), *M*=36.11% (*SD*=36.38, 0-100), and *M*=31.28% (*SD*=35.13, 0-100) of their patients of potential childbearing age, about a possible association between hormonal contraception intake and target, other psychological and other somatic symptoms respectively. Generally, it was considered as important for the psychotherapist to know if a patient was affected by hormonal contraception-related onset or worsening of target symptoms (affected patients: *M*=95.1, *SD*=12.65, 60-100; psychotherapists: *M*=72.28, *SD*=31.57, 0-100), other psychological symptoms (affected patients: *M*=85.57, *SD*=16.81, 56-100; psychotherapists: *M*=67.18, *SD*=31.57, 0-100) and other somatic symptoms(affected patients: *M*=58.27, *SD*=34.02, 0-100; psychotherapists: *M*=57.5, *SD*=33.47, 0-100), respectively.


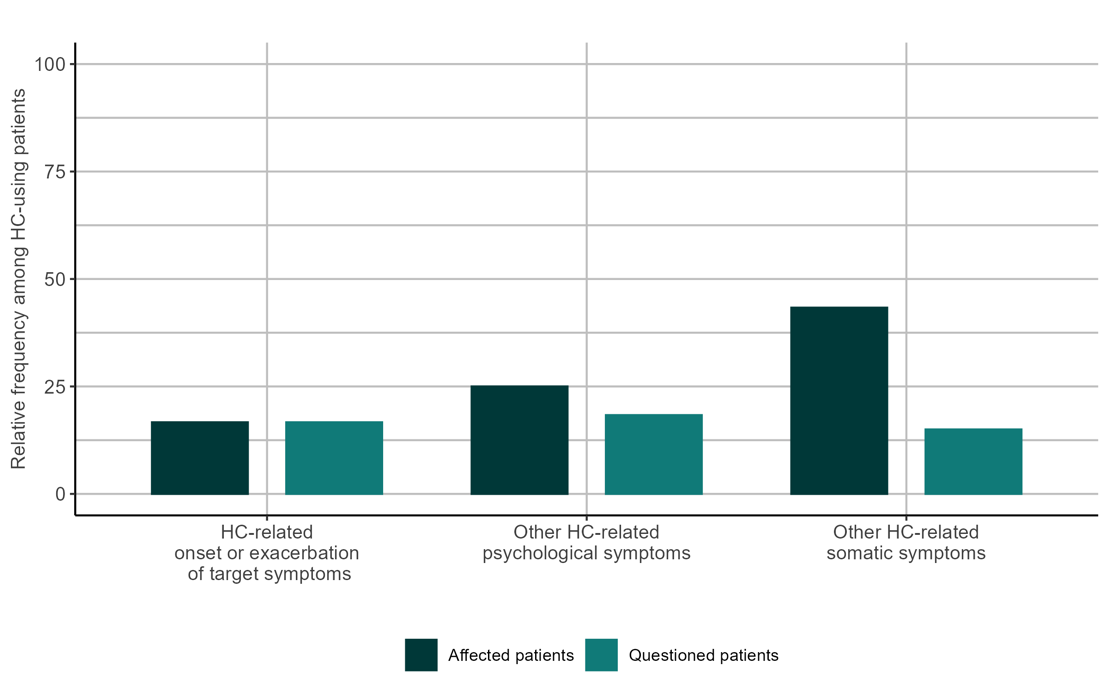


Supplementary figure S3. Discrepancy between number of patients affected by hormonal contraception (HC)-related onset or worsening of target symptoms, other psychological, and other physical symptoms, versus patients asked about this.

**Pregnancy**

*Detailed methods: assessment of pregnancy-related aspects.* We further asked patients who reported that they had already experienced one or more pregnancies whether they had given birth to one or more children (referring to live births, as opposed to miscarriages), whether they had experienced one or more miscarriages, and whether they had received one or more abortions. Further, patients were asked to indicate whether the target, other psychological or other somatic symptoms first occurred or worsened during a pregnancy. They were also asked whether they had been pregnant unplanned, unintended or unwanted and whether they had experienced medical complications and medical interventions during a pregnancy. Analogous to the methodology described in the manuscript, we also asked patients whether they had been proactively been asked about these aspects, we asked psychotherapists how often they proactively asked about these aspects, and we further asked both participant groups about the perceived relevance of these aspects for psychotherapy.

*Detailed results: prevalence, consideration and relevance of pregnancy-related aspects.* As noted in the manuscript, *n*=71 (24.4%) patients reported that they had already been pregnant. Of those, *n*=46 (64.79%) patients indicated that they had given birth to one or more children. n=57 (80.28%) patients indicated that they had been asked about this by their psychotherapist and psychotherapists reported that they asked *M*=74.98% (*SD*=38.84, 0-100) in patients with a uterus about live births. 24 (33.8%) and *n*=13 (18.31%) patients who had been pregnant indicated that they had experienced a miscarriage and received an abortion, respectively, but only *n*=20 (28.17%) and *n*=9 (12.68%) had been asked about this in psychotherapy. Psychotherapists reported that they proactively asked *M*=52.83% (*SD*=42.16, 0-100) and *M*=43.17% (*SD*=42.43, 0-100) of their patients with a uterus about past miscarriages and abortions, respectively. At the same time, both aspects were considered as highly relevant for psychotherapy (miscarriage, affected patients: *M*=85.58, *SD*=23.96, 13-100; miscarriage, psychotherapists: *M*=86.66, *SD*=24.01, 0-100; abortion, affected patients: *M*=77.5, *SD*=34.53, 15.100; abortion, psychotherapists: *M*=83.02, *SD*=25.81, 0-100).

Among patients who had been pregnant, *n*=26 (36.62%) reported an association between their pregnancies and their target symptoms, while *n*=29 (40.85%) reported an association with other psychological and *n*=41 (57.85%) with other somatic symptoms. However, only *n*=22 (30.99%), *n*=18 (25.35%) and *n*=19 (26.76%) were asked about target, other psychological and other somatic symptoms related to their pregnancies, respectively. Psychotherapists reported that they proactively asked *M*=46.8% (*SD*=39.21, 0-100), *M*=48.94% (*SD*=38.91, 0-100), and *M*=44.37% (*SD*=38.45, 0-100) of their patients with a uterus, about possibly pregnancy-related target, other psychological and other somatic symptoms, respectively. Patients and psychotherapists considered as important for the psychotherapist to know if target symptoms (affected patients: *M*=88.88, *SD*=16.45, 35-100; psychotherapists: *M*=85.82, *SD*=24.18, 0-100), other psychological symptoms (affected patients: *M*=87.48, *SD*=15.54, 36-100; psychotherapists: *M*=82.75, *SD*=25.21, 0-100) and other somatic symptoms (affected patients: *M*=66.59, *SD*=27.86, 0-100; psychotherapists: *M*=69.86, *SD*=31.05, 0-100), were related to a pregnancy, respectively.

26 (36.62%) of patients who had been pregnant reported that one or more of these pregnancies were unplanned, unintended or unwanted. Psychotherapists reported that they asked *M*=51.72% (*SD*=35.58, 0-100) of patients who had been pregnant pregnancy intention. Again, patients and psychotherapists considered it important for the psychotherapist to know about an unplanned, unintended or unwanted pregnancy (affected patients: *M*=81.28, *SD*=26.83, 18-100; psychotherapists: *M*=85.2, *SD*=23.42, 0-100). Lastly, *n*=21 (29.58%) and n=6 (8.45%) patients who had been pregnant had experienced medical complications and interventions during a pregnancy, respectively, and *n*=24 (33.8%) and *n*=9 (12.68%) had been asked about this during psychotherapy. Psychotherapists reported that they asked *M*=54.66% (*SD*=42.16, 0-100) and *M*=42.83% (*SD*=39.84, 0-100) of their patients with a uterus, about potential medical complications and interventions during pregnancy, respectively. However, medical complications and interventions were also considered as relevant for psychotherapy (medical complications, affected patients: *M*=76, *SD*=31.76, 5-100; medical complications, psychotherapists: *M*=77.14, *SD*=22.54, 0-100; medical interventions, affected patients: *M*=72, *SD*=28.01, 24-100; medical interventions, psychotherapists: *M*=71.02, *SD*=30.05, 0-100).


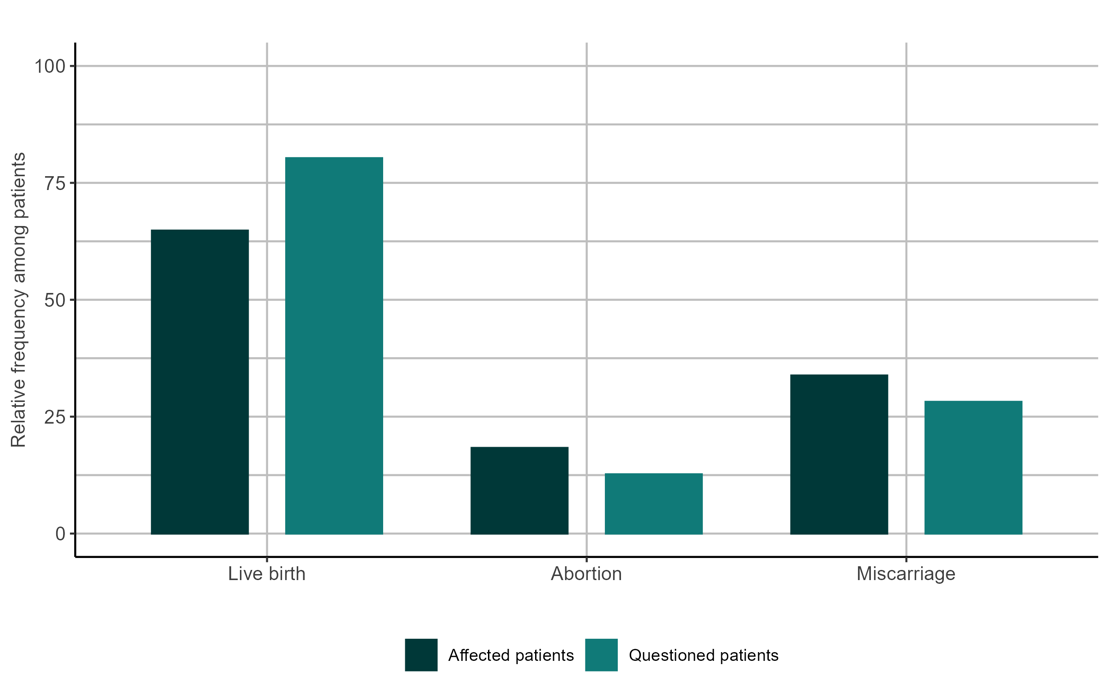


Supplementary figure S4. Discrepancy between number of patients who experienced a live birth, abortion, or miscarriage, versus patients asked about this.

**
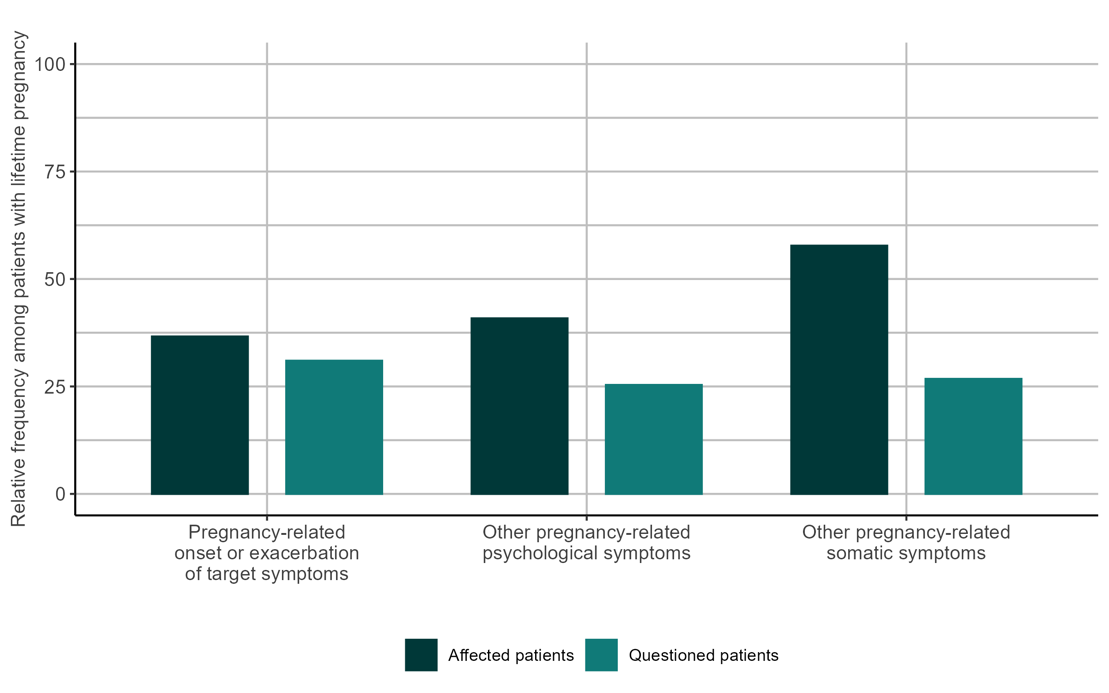
**

Supplementary figure S5. Discrepancy between number of patients who experienced pregnancy-related onset or worsening of target symptoms, other psychological, and other physical symptoms, versus patients asked about this.

**
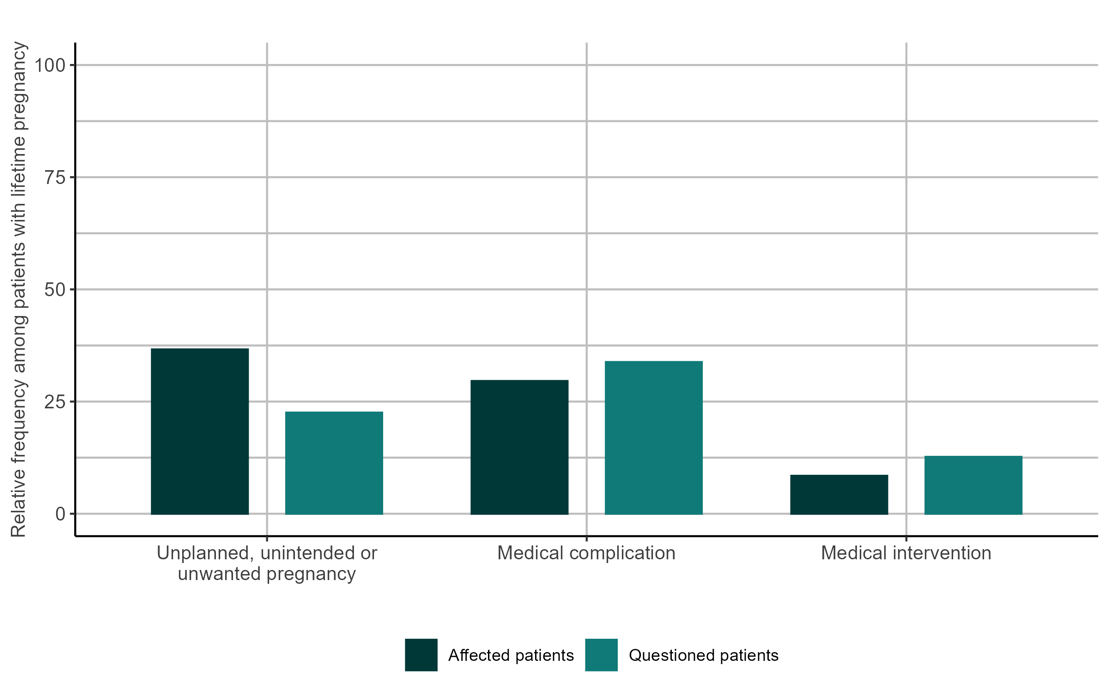
**

Supplementary figure S6. Discrepancy between number of patients who experienced an unplanned, unintended, or unwanted pregnancy, medical complication during pregnancy, or medical intervention during pregnancy, versus patients asked about this.

**Fertility treatment**

*Detailed methods: assessment of fertility treatment-related aspects.* We asked patients whether they had a desire to have one or more (additional) biological children. We further asked patients whether they had received fertility treatment. Those who indicated that they had were asked whether the psychotherapeutic target symptoms had first occurred or worsened during fertility treatment, as well as for other psychological symptoms and other somatic symptoms related to fertility treatment (response format: ‘yes’/’no’/ ‘I do not know’/ ‘no response’). Analogous to the methodology described in the manuscript, we also asked patients whether they had been proactively been asked about these aspects, we asked psychotherapists how often they proactively asked about these aspects, and we further asked both participant groups about the perceived relevance of these aspects for psychotherapy.

*Detailed results: prevalence, consideration and relevance of fertility treatment-related aspects.* 89 (30.58%) patients indicated that they desired to have one or more (additional) biological children, *n*=98 (33.68%) reported that their psychotherapist had asked about this. Psychotherapists reported that they asked *M*=58.59% (*SD*=36.36, 0-100) of their patients with a uterus about their potential desire for one or more (additional) biological children. Patients and psychotherapists considered it important for the psychotherapist to know if a patient had an unfulfilled desire to have one or more (additional) biological children (affected patients: *M*=82.15, *SD*=27.71, 0-100; psychotherapists: *M*=78.47, *SD*=31.24, 0-100). As noted in the manuscript, *n*=16 (5.5%) patients stated that had received fertility treatment. Of those, *n*=6 (8.45%) reported an association between fertility treatment and their target symptoms, *n*=5 (7.04%) reported an association with other psychological and *n*=8 (11.27%) with other somatic symptoms. Only *n*=4 (5.63%), *n*=5 (7.04%) and *n*=2 (2.82%) were asked about target, other psychological and other somatic symptoms related to fertility treatment, respectively. Psychotherapists reported that they proactively asked *M*=46.48% (*SD*=42.82, 0-100), *M*=49.88% (*SD*=42.53, 0-100), and *M*=42.69% (*SD*=41.9, 0-100) of their patients with a uterus, about potential associations between their target, other psychological, and other somatic symptoms, respectively. Generally, it was considered as important for the psychotherapist to know if a patient was affected by fertility treatment-related onset or worsening of target symptoms (affected patients: *M*=100, *SD*=0, 100-100; psychotherapists: *M*=83.86, *SD*=27.57, 0-100), other psychological symptoms (affected patients: *M*=100, *SD*=0, 100-100; psychotherapists: *M*=80.38, *SD*=29.03, 0-100) and other somatic symptoms (affected patients: *M*=90.88, *SD*=13.2, 70-100; psychotherapists: *M*=69.15, *SD*=32.8, 0-100), respectively.

**Birth**

*Detailed methods: assessment of birth-related aspects.* We asked patients who reported that they had experienced one or more live births whether they had experienced medical complications or interventions during birth and whether they perceived their birth as distressing. Further, they were asked to indicate whether the target, other psychological or other somatic symptoms first occurred or worsened during the puerperium. Analogous to the methodology described in the manuscript, we also asked patients whether they had been proactively been asked about these aspects, we asked psychotherapists how often they proactively asked about these aspects, and we further asked both participant groups about the perceived relevance of these aspects for psychotherapy.

*Detailed results: prevalence, consideration and relevance of birth-related aspects.* As noted in the manuscript, *n*=46 (15.81%) patients had experienced a live birth. Of those, *n*=28 (60.87%) and *n*=26 (56.52%) had experienced medical complications and interventions during birth, respectively, but only *n*=22 (47.83%) and *n*=19 (26.67%) had been asked about this during psychotherapy. Psychotherapists reported average proactive inquiry of *M*=51.8 (*SD*=40.31, 0-100) and *M*=41.96 (*SD*=4.15, 0-100) in patients with a uterus, respectively. However, medical complications and interventions were considered as relevant for psychotherapy (medical complications, affected patients: *M*=80.29, *SD*=30, 0-100; medical complications, psychotherapists: *M*=76.02, *SD*=29.12, 0-100; medical interventions, affected patients: *M*=68.23, *SD*=36.3, 0-100; medical interventions, psychotherapists: *M*=71.79, *SD*=30.27, 0-100). 28 (60.87%) patients perceived birth as distressing, and 22 (47.83%) reported that their psychotherapist asked about this. Psychotherapists indicated to ask *M*=53.86% (*SD*=39.88, 0-100) of their patients with a uterus about distressing birth experiences, while it was generally considered as important for psychotherapists to know about distressing birth experiences (affected patients: *M*=87.93, *SD*=26.33, 0-100; psychotherapists: *M*=88.43, *SD*=22.76, 0-100).

24 (52.xx17 *n*=23 (50 %) and *n*=24 (52.17%) patients who had experienced a live birth indicated that their target symptoms, other psychological and other somatic symptoms first occurred or worsened during the puerperium. However, only *n*=18 (39.13%), *n*=18 (39. 13%) and *n*=18 (39. 13%) were asked about this respectively. Psychotherapists indicated that they asked *M*=53.53% (*SD*=39.17, 0-100), *M*=47.8% (*SD*=39.79, 0-100), and *M*=40.2% (*SD*=38.49, 0-100) of their patients who had experienced a live birth about onset or worsening of target, other psychological and other somatic symptoms during the puerperium, respectively. At the same time, both groups of participants considered it as important for the psychotherapist to know if a patient was affected by puerperium-related onset or worsening of target symptoms (affected patients: *M*=89.33, *SD*=20.63, 20-100; psychotherapists: *M*=85.43, *SD*=24.79, 0-100), other psychological symptoms (affected patients: *M*=83.48, *SD*=28.04, 5-100; psychotherapists: *M*=81.62, *SD*=26.59, 0-100) and other somatic symptoms (affected patients: *M*=63.61, *SD*=36.16, 5-100; psychotherapists: *M*=69, *SD*=31.69, 0-100), respectively.


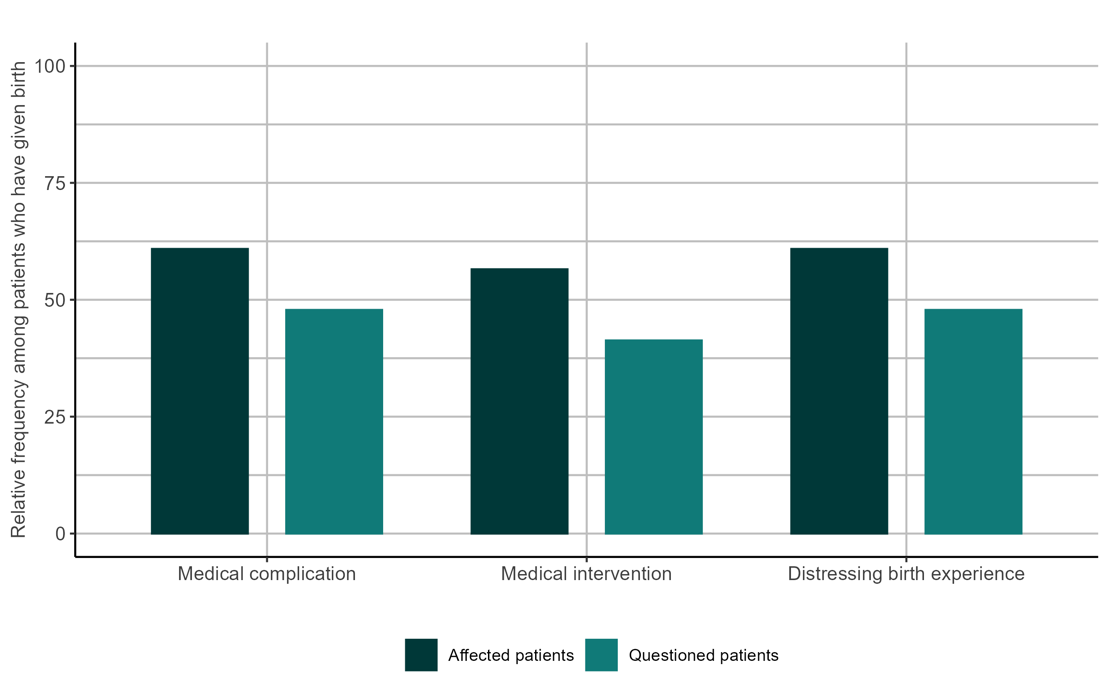


Supplementary figure S7. Discrepancy between number of patients who experienced a medical complication during birth, medical intervention during birth, or distressing birth, versus patients asked about this.

**
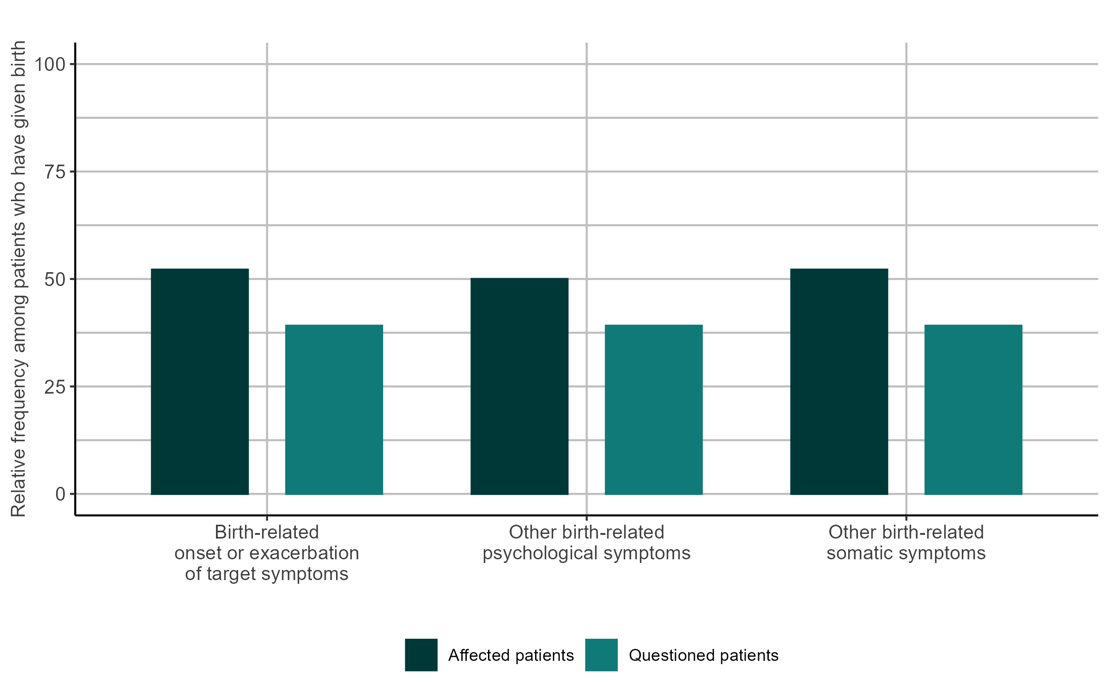
**

Supplementary figure S8. Discrepancy between number of patients who experienced a childbirth-related onset or worsening of target symptoms, other psychological, and other physical symptoms, versus patients asked about this.
