# Supplementary material for: Reproductive health – a blind spot in psychotherapeutic treatment? Evidence of insufficient consideration of reproductive factors in routine care
Source: Dialogues Clin Neurosci. 2026 Apr 19;28(1):157–65. doi: 10.1080/19585969.2026.2653598 (PMC13094238; doi:10.1080/19585969.2026.2653598)
Supplement: Sup3_2025_11_29.docx [file TDCN_A_2653598_SM4584.docx]

**Supplementary material 3**

**Participant information**

**Table 1**

|  |  | Psychotherapists (*n* = 390) | Patients (*n* = 291) | |
| --- | --- | --- | --- | --- |
| Age | | M = 45.18 (SD = 10.49) |  | |
|  | 18 - 24 |  | n = 81 (27.84%) | |
|  | 25 - 30 |  | n = 99 (34.02%) | |
|  | 31 - 40 |  | n = 80 (27.49%) | |
|  | 41 - 50 |  | n = 25 (8.59%) | |
|  | 51 - 60 |  | n = 4 (1.37%) | |
|  | 61- 70 |  | n = 2 (0.69%) | |
|  | older than 70 |  | - | |
| Gender^(a)^ | |  |  | |
|  | Female | n = 343 (87.95%) | n = 278 (95.53%) | |
|  | Male | n = 44 (11.28%) | n = 3 (1.03%) | |
|  | Cis | n = 45 (11.54%) | n = 48 (16.49%) | |
|  | Endo* | - | n = 1 (0.34%) | |
|  | Inter* | - | - | |
|  | Non-binary | - | n = 14 (4.81%) | |
|  | Questioning | n = 1 (0.26%) | n = 13 (4.47%) | |
|  | Trans* | - | n = 5 (1.72%) | |
|  | Preferred self-identification | n = 6 (1.5%) | n = 5 (1.72%) | |
|  | Chose not to answer | n = 2 (0.5%) | n = 2 (0.69%) | |
| Sex | |  |  | |
|  | Female | n= 342 (87.69%) | n = 290 (99.66%) | |
|  | Male | n = 45 (11.54%) | n = 1 (0.34%) | |
|  | Unknown | n = 1 (0.26%) | - | |
|  | Chose not to answer | n = 2 (0.51%) | - | |
| License | |  |  | |
|  | Full license | n = 378 (96.92%) |  | |
|  | In training | n = 13 (3.33%) |  | |
| Psychotherapeutic approaches^(a)^ | |  |  | |
|  | Analytic psychotherapy | n = 15 (3.85%) | n = 11 (3.78%) | |
|  | Cognitive behavioral therapy | n = 328 (84.1%) | n = 186 (63.92%) | |
|  | Depth-psychology based psychotherapy | n = 66 (16.92%) | n = 83 (28.52%) | |
|  | Systemic psychotherapy | n = 3 (0.77%) | n = 11 (3.78%) | |
| Diagnoses^(a)^ | |  |  | |
|  | Mood (affective) disorders | n = 381 (97.69%) | n = 200 (68.73%) | |
|  | Anxiety disorders | n = 387 (99.23%) | n = 118 (40.55%) | |
|  | Obsessive-compulsive disorders | n = 336 (86.15%) | n = 16 (5.5%) | |
|  | Somatoform disorders | n = 345 (88.46%) | n = 21 (7.22%) | |
|  | Dissociative (conversion) disorders | n = 198 (50.77%) | n = 25 (8.59%) | |
|  | Reaction to severe stress, and adjustment disorders | n = 374 (95.50%) | n = 118 (40.55%) | |
|  | Eating disorders | n = 287 (73.59%) | n = 50 (17.18%) | |
|  | Nonorganic sleep disorders | n = 238 (61.03%) | n = 12 (4.12%) | |
|  | Sexual dysfunction | n = 175 (44.87%) | n = 3 (1.03%) | |
|  | Disorders of adult personality | n = 343 (87.95%) | n = 28 (9.62%) | |
|  | Disorders of adult behavior | n = 252 (64.62%) | n = 6 (2.06%) | |
|  | Behavioral and emotional disorders with onset usually occuring in childhood and adolescence | n = 200 (51.28%) | n = 30 (10.31%) | |
|  | (Behavioral) disorders associated with psychotropic drugs | n = 145 (37.18%) | n = 4 (1.37%) | |
|  | Schizophrenic disorders | n = 143 (36.67%) | n = 2 (0.69%) | |
|  | Developmental disorders | n = 9 (2.31%) | n = 2 (0.69%) | |
|  | Conditions related to sexual health | n = 8 (2.05%) | n = 1 (0.34%) | |
|  | Psychological and behavioral factors associated with disorders/diseases classified elsewhere | n = 5 (1.28%) | n = 2 (0.69%) | |
|  | Psychological and behavioral factors associated with gynecological diseases/conditions | n = 2 (0.51%) | n = 4 (1.37%) | |
|  | Not mentioned above | n = 2 (0.51%) | n = 2 (0.69%) | |
|  | Chose not to answer | - | n = 3 (1.03%) | |
| Therapeutic setting ^(a)^ | |  |  | |
|  | Outpatient setting | n = 384 (98.46%) | n = 275 (94.5%) | |
|  | Day-care | n = 4 (1.03%) | n = 13 (4.47%) | |
|  | Inpatient setting | n = 114 (29.23%) | n = 29 (9.97%) | |
|  | Other | n = 3 (0.77%) | n = 4 (1.37%) | |
| Number of completed treatments | |  |  | |
|  | 11 - 20 | n = 6 (1.54%) |  | |
|  | 21 - 50 | n= 56 (14.36%) |  | |
|  | 51 - 100 | n = 80 (20.51%) |  | |
|  | 101 - 200 | n = 80 (20.51%) |  | |
|  | 201 - 500 | n = 94 (24.1%) |  | |
|  | > 500 | n = 69 (17.69%) |  | |
| Number of completed sessions^(b)^ | |  | M = 51.37 (SD = 53.51) | |
| ^(a)^allowed for multiple choice | |  | |  |
| ^(b)^two outliers (5050, 1000 sessions) were removed as data seemed highly implausible | | | | |
