# Supplementary material for: Reproductive health – a blind spot in psychotherapeutic treatment? Evidence of insufficient consideration of reproductive factors in routine care
Source: Dialogues Clin Neurosci. 2026 Apr 19;28(1):157–65. doi: 10.1080/19585969.2026.2653598 (PMC13094238; doi:10.1080/19585969.2026.2653598)
Supplement: Sup2_2025_07_19.docx [file TDCN_A_2653598_SM4583.docx]

**Supplementary material 2**

**Information about the reproductive factors/aspects provided in the survey for psychotherapists**

**Menstrual cycle**

The first part of this survey is about the menstrual cycle. This reproductive factor affects individuals from menarche, i.e. the first menstrual bleeding at an average age of 13 years, to menopause, i.e. the time of the last menstruation at an average age of 51 years. The menstrual cycle lasts around 28 days. Cycle lengths of between 25 and 35 days are considered as normal. Day 1 marks the start of menstrual bleeding, which lasts an average of five days, and the beginning of the follicular phase. After around 14 days, ovulation occurs, which can be determined using special hormone tests and symptom observations (e.g. a rise in basal body temperature). A this timepoint, pregnancy can occur. Ovulation is followed by the luteal phase, which lasts an average of 14 days. Fluctuations in cycle length or temporarily absent cycles occur in particular after menarche, during the menopausal transition, during and after pregnancies or due to illness and stress.

**Hormonal contraception**

The following part of the survey is about hormonal contraception. The most commonly used method is the pill, which is used by 38% of individuals between the ages of 20 and 44. Other common methods include the hormonal coil, the vaginal ring, the three-month or depot injection and the contraceptive or hormonal patch. In different doses and via different routes, all of these methods release the hormones progestogen and/or oestrogen into the body, thus suppressing the functions of the menstrual cycle and preventing pregnancies. Non-hormonal contraceptive methods that do not affect the menstrual cycle include condoms, the copper IUD and natural family planning methods such as the symptothermal method.

**Pregnancy**

The following part of the survey is about pregnancy. From the first day of the last menstrual period to a live birth, the duration of a pregnancy is on average 40 weeks. A pregnancy can be divided into three trimesters, each lasting about three months. Not every pregnancy ends in a live birth: around 30% of pregnancies end in miscarriage, particularly frequently in the first trimester. Not all pregnancies are wanted: around 17% of women aged between 20 and 44 have had an unintended pregnancy at least once in their lives. Over 8% of women of childbearing age have already had an abortion. In Germany, abortion is a criminal offense that is not being punished under certain conditions. Between 13% and 25% of German women who do not have children of their own state that they are unintentionally childless, many of them due to their reproductive health. The number of fertility treatments has been increasing for years. Common interventions include insemination, in-vitro fertilization or intratubal gamete transfer. These interventions are almost always preceded by treating the individual who wants to become pregnant with hormones.

**Childbirth**

The following part of the survey is about childbirth. Childbirth can be spontaneous and vaginal or it can be accompanied by various medical interventions, e.g. medications to induce or stimulate labor, forceps or a suction cup. More than 30% of births in Germany are caesarean sections. Live births are followed by the postpartum period. The first eight weeks after a birth (“puerperium”) are characterized by hormonal changes as well as changes in roles. Around 77% of mothers breastfeed their child during this time, which is also associated with hormonal processes.
